# Online Health Information–Seeking in the Era of Large Language Models: Cross-Sectional Web-Based Survey Study

**DOI:** 10.2196/68560

**Published:** 2025-03-31

**Authors:** Hye Sun Yun, Timothy Bickmore

**Affiliations:** 1 Khoury College of Computer Sciences Northeastern University Boston, MA United States

**Keywords:** online health information–seeking, large language models, eHealth, internet, consumer health information

## Abstract

**Background:**

As large language model (LLM)–based chatbots such as ChatGPT (OpenAI) grow in popularity, it is essential to understand their role in delivering online health information compared to other resources. These chatbots often generate inaccurate content, posing potential safety risks. This motivates the need to examine how users perceive and act on health information provided by LLM-based chatbots.

**Objective:**

This study investigates the patterns, perceptions, and actions of users seeking health information online, including LLM-based chatbots. The relationships between online health information–seeking behaviors and important sociodemographic characteristics are examined as well.

**Methods:**

A web-based survey of crowd workers was conducted via Prolific. The questionnaire covered sociodemographic information, trust in health care providers, eHealth literacy, artificial intelligence (AI) attitudes, chronic health condition status, online health information source types, perceptions, and actions, such as cross-checking or adherence. Quantitative and qualitative analyses were applied.

**Results:**

Most participants consulted search engines (291/297, 98%) and health-related websites (203/297, 68.4%) for their health information, while 21.2% (63/297) used LLM-based chatbots, with ChatGPT and Microsoft Copilot being the most popular. Most participants (268/297, 90.2%) sought information on health conditions, with fewer seeking advice on medication (179/297, 60.3%), treatments (137/297, 46.1%), and self-diagnosis (62/297, 23.2%). Perceived information quality and trust varied little across source types. The preferred source for validating information from the internet was consulting health care professionals (40/132, 30.3%), while only a very small percentage of participants (5/214, 2.3%) consulted AI tools to cross-check information from search engines and health-related websites. For information obtained from LLM-based chatbots, 19.4% (12/63) of participants cross-checked the information, while 48.4% (30/63) of participants followed the advice. Both of these rates were lower than information from search engines, health-related websites, forums, or social media. Furthermore, use of LLM-based chatbots for health information was negatively correlated with age (ρ=–0.16, *P*=.006). In contrast, attitudes surrounding AI for medicine had significant positive correlations with the number of source types consulted for health advice (ρ=0.14, *P*=.01), use of LLM-based chatbots for health information (ρ=0.31, *P*<.001), and number of health topics searched (ρ=0.19, *P*<.001).

**Conclusions:**

Although traditional online sources remain dominant, LLM-based chatbots are emerging as a resource for health information for some users, specifically those who are younger and have a higher trust in AI. The perceived quality and trustworthiness of health information varied little across source types. However, the adherence to health information from LLM-based chatbots seemed more cautious compared to search engines or health-related websites. As LLMs continue to evolve, enhancing their accuracy and transparency will be essential in mitigating any potential risks by supporting responsible information-seeking while maximizing the potential of AI in health contexts.

## Introduction

Searching for health information remains one of the most common uses of the internet. Over the last decade, multiple surveys have demonstrated that most adults use the internet for health and medical information [1-4], with most respondents reporting that they turn to the internet as their first source of health information [1]. Adults use the internet for a range of health-related information, including general information on health-related topics, such as diet and exercise, or information on specific injuries or diseases and their treatment, or for self-diagnosis [5].

While search engines remain the primary means of finding medical advice on the internet [5], people are increasingly turning to other channels—including health-related websites, such as WebMD, and social media—for health information [6]. With the availability and explosion in the use of large language model (LLM)–based chatbots, such as ChatGPT, Bard (Google LLC), and Claude (Anthropic), preliminary evidence suggests that these chatbots are becoming increasingly popular for obtaining medical advice [6,7]. A recent survey of US adults found that 17% of respondents reported using chatbots at least once per month to find health information (25% for adults aged younger than 30 years) [6].

The rapid rise of LLM-based chatbots presents both opportunities and concerns. Despite their potential, these chatbots are known to generate inaccurate or misleading information. Evaluations of LLM-based chatbot accuracy in answering medical queries—while improving—have shown their accuracy to be as low as 4% [8-15]. Given these safety risks, a critical question remains: how do users perceive and act upon medical advice obtained from LLM-based chatbots? Although there are no studies to date on the rate at which laypersons actually follow medical advice from an LLM-based chatbot, studies have found that many users report blindly following medical advice they found via internet search engines. A 2013 survey found that 35% of US respondents reported using the internet to diagnose a condition, and, of these, 35% indicated they did not follow up with a clinician to confirm their findings [16]. More recent studies show a continued reliance on online sources, with 58.5% of United States and 65.3% of French adults reporting that they search for medical information online [2,17]. Alarmingly, other surveys found that 7%-78% of respondents said they were willing to use ChatGPT, as-is, for self-diagnosis for a medical condition [18,19]. Furthermore, prior research suggests that users may trust ChatGPT more than traditional search engines in health-related contexts, with user-friendly features and prior experience influencing trust in ChatGPT and their outputs [20]. However, such studies have often been limited by small sample sizes and controlled laboratory settings.

Existing research has largely focused on the prevalence of health information–seeking behaviors online but has not sufficiently addressed what users do with the medical advice they obtain, especially from LLM-based chatbots. While some studies suggest that users may blindly follow online health advice, the extent to which chatbot-generated information is trusted or acted upon has not been adequately investigated. This lack of understanding presents a critical gap in the literature, as the potential harm from acting on inaccurate chatbot advice remains underexplored.

To address this gap, this study aims to examine the prevalence of LLM-based chatbot use for medical advice in comparison to other online sources and explore how users perceive and act upon this advice. We also go beyond this to investigate the trust respondents place in this advice, their subsequent actions to cross-check and validate the information received, and their intent to follow the advice. These insights provide a deeper understanding of health decision-making pathways and the potential risks associated with reliance on LLM-based chatbots for medical information.

## Methods

### Study Design and Recruitment

This study was a cross-sectional, anonymous, self-administered questionnaire survey with consecutive sampling. All participants were recruited through the Prolific web-based research platform regardless of their LLM-based chatbot usage [21]. The inclusion criteria were the following: (1) aged 18 years or older and (2) English as a primary language. We excluded participants who failed to correctly respond to an “attention check” question in the survey, as is common practice in web-based studies [22-24]. We intentionally recruited participants internationally to better understand how users from different countries seek health information online. A total of 300 participants who met the inclusion criteria were recruited to fill out the survey in May 2024. Given a prior study’s finding that 17% of respondents reported using LLM-based chatbots for health information [6], we determined that a sample size of 300 would provide a margin of error of ±4% for this and similar measures of proportion, which was adequate for our analyses.

Web-based questionnaires were distributed in English to the participants using a Qualtrics web survey. The survey took approximately 15 minutes to complete.

### Ethical Considerations

Ethics approval was received from the Institutional Review Board at Northeastern University (reference 24-5-05). All participants completed an informed consent process, which allowed them to opt out of any survey questions or withdraw from the study at any time. The participants were compensated US $3 after completing the survey. All collected data was deidentified.

### Survey Measures

Our questionnaire was developed based on a review of existing literature on online health information–seeking behavior. The final questionnaire (Multimedia Appendix 1) covered sociodemographic information, diagnosed chronic health status, familiarity and usage of ChatGPT, the full eHealth Literacy Scale (eHEALS) [25], Trust in the Health Care Team (T-HCT) Scale [26], the 4-item Artificial Intelligence Attitude Scale (AIAS-4) [27], and items related to online health information–seeking behavior over the past year.

The eHEALS is one of the most widely used validated measures of eHealth literacy and has been validated with various population groups [28-31]. eHEALS contains 8 questions on a 5-point Likert scale, measuring various aspects of self-perceived eHealth literacy. The composite score involves the sum of all items (range 8-40), with higher scores indicating higher eHealth literacy.

The T-HCT Scale consists of 29 items on a 5-point Likert scale and assesses the trust of participants in health care teams. We averaged all items for the composite measure. Furthermore, AIAS-4 was used to understand participants’ trust in artificial intelligence (AI) technology. AIAS-4 contains 4 items on a 10-point Likert scale, with the averaging of all items providing the composite measure. In the survey, we additionally included a separate item in the same format as AIAS-4 regarding the benefits of AI in medicine.

Most importantly, our survey asked participants about their online health information–seeking behavior over the last year and their trust in and perceptions of the information they found online. We asked participants to indicate all the tools they have used for online health information in the past year, including (1) search engines (Google, Bing [Microsoft Corp], Yahoo, etc), (2) social media (Twitter [X Corp], Facebook [Meta], Instagram [Instagram from Meta], Reddit, etc), (3) health community forums, (4) health-related websites (eg, WebMD, Mayo Clinic, PubMed Central), (5) LLM-based chatbots (eg, ChatGPT, Bard, Gemini [Google LLC], Microsoft Copilot, YouChat, Perplexity AI, ErnieBot [Baidu, Inc]), (6) conversational assistants (eg, Siri [Apple Inc], Alexa [Amazon.com, Inc], and Google Home), (7) health apps (eg, diagnosis tool), and (8) other sources. For each of the tools used in the past year, we asked what type of health information they searched for and their assessments of the information received along the dimensions of accuracy, satisfaction, helpfulness, trustworthiness, usefulness, ease of understanding, and feelings of reduced anxiety, on 5-point Likert scale (from “strongly disagree” to “strongly agree”). Finally, we asked if they took any actions to cross-check or follow the health advice provided to them. Participants had the option to provide an open response on how they cross-checked the information. The exact wording of these items can be found in Multimedia Appendix 1.

### Statistical Analysis

Descriptive statistics were computed to summarize the data, with means and SDs calculated where applicable. A bivariate analysis was performed between the independent variables, such as the number of online source types consulted, and the dependent variables such as age or eHEALS. Data were analyzed using R (version 4.3.0; R Foundation for Statistical Computing) in R Studio (version 2023.06.1+524).

Additionally, we conducted an inductive thematic analysis of the open-ended responses (3335 words), guided by sensitizing concepts that focused on how participants cross-check the health information they find online [32]. We used elements of the grounded theory method (open, axial, and selective coding) [33]. The qualitative analysis was carried out using NVivo (version 14.23.0; Lumivero).

## Results

The total number of survey respondents was 300. Only 1% (3/300) of participants failed the attention-checking question. We removed the data from these participants before our analysis.

### Sociodemographic Characteristics of Participants

Table 1 shows the sociodemographic characteristics of the survey participants. The ages of the participants ranged from 18 to 78 years (mean 36, SD 12.7 years). Most participants identified as male (184/295, 62.4%), were White (190/296, 64.2%), were not diagnosed with chronic disease (199/287, 69.3%), and spoke English as a first language (242/297, 81.5%). Participants reported 10-26 years for the total number of years they have completed primary or elementary, secondary, or postsecondary education (mean 16, SD 2.9). Most participants (120/296, 40.5%) self-reported their average household net adjusted disposable income to be around the median compared to OECD’s (Organisation for Economic Co-Operation and Development) Better Life Index statistics of their respective country of residence [34]. The top represented countries of residence were the United States (69/297, 23.2%), United Kingdom (54/297, 18.2%), Canada (43/297, 14.5%), Australia (30/297, 10.1%), South Africa (26/297, 8.8%), and Poland (14/297, 4.7%). The mean T-HCT score of participants was 3.5 (SD 0.6), with the mean eHEALS score being 30.8 (SD 4.7) and the mean AIAS-4 score being 6.5 (SD 2.3). Most participants perceived AI to be beneficial in medicine, with a median value of 7 (IQR 4) on a 10-point Likert scale.

**Table 1:** Sociodemographic characteristics of survey participants.

Characteristics			Participants, n (%)		Total number
**Age (years)**					297
	18-24	48 (16.2)			
	25-34	113 (38)			
	35-44	71 (23.9)			
	45-54	36 (12.1)			
	≥55	29 (9.8)			
**Sex**					295
	Female	111 (37.6)			
	Male	184 (62.4)			
**Race**					296
	Asian	36 (12.2)			
	Black	36 (12.2)			
	White	190 (64.2)			
	Mixed	23 (7.8)			
	Other	11 (3.7)			
**Country of residence**					297
	United States	69 (23.2)			
	United Kingdom	54 (18.2)			
	Canada	43 (14.5)			
	Australia	30 (10.1)			
	South Africa	26 (8.8)			
	Poland	14 (4.7)			
	Mexico	9 (3)			
	Portugal	9 (3)			
	Germany	6 (2)			
	Other	37 (12.5)			
**Occupation**					293
	Managers, professionals, and academic staff	95 (32.4)			
	Homemakers, retired, and unemployed	45 (15.4)			
	Tech (IT^a^) workers	42 (14.3)			
	Clerics, services, and sales workers	36 (12.3)			
	Craft workers and laborers	28 (9.6)			
	Student	22 (7.5)			
	Technicians and associate professionals	18 (6.1)			
	Health care workers	7 (2.4)			
**Education (years)**					295
	10-12	40 (13.6)			
	13-16	150 (50.8)			
	≥17	105 (35.6)			
Have a diagnosed chronic medical condition			88 (30.7)		287
**Average household net income**					296
	Extremely below the median	30 (10.1)			
	Somewhat below the median	59 (19.9)			
	Around the median	120 (40.5)			
	Somewhat above the median	78 (26.4)			
	Extremely above the median	9 (3)			
**T-HCT^b^ mean score (range 1-5; mean 3.5, SD 0.6)**					297
	<3.5	134 (45.1)			
	≥3.5	163 (54.9)			
**eHEALS^c^ sum score (range 8-40; mean 30.8, SD 4.7)**					297
	<31	120 (40.4)			
	≥31	177 (59.6)			
**AIAS-4^d^ mean score (range 1-10; mean 6.5, SD 2.3)**					297
	<6.75	143 (48.1)			
	≥6.75	154 (51.9)			
**Perceptions of AI^e^ to be beneficial in medicine (range 1-10)**					297
	<7	109 (36.7)			
	≥7	188 (63.3)			

^a^IT: information technology.

^b^T-HCT: Trust in the Health Care Team.

^c^eHEALS: eHealth Literacy Scale.

^d^AIAS-4: 4-Item Artificial Intelligence Attitude Scale.

^e^AI: artificial intelligence.

### Sources of Online Health Information Seeking

All participants reported seeking health information or advice online in the prior year using various online sources (Table 2). Most participants consulted search engines (eg, Google, Bing, Yahoo; 291/297, 98%) and health-related websites (eg, WebMD, Mayo Clinic, PubMed Central; 203/297, 68.4%). Less popular sources were social media (eg, Twitter, Facebook, Instagram, Reddit; 119/297, 40.1%), health community forums (84/297, 28.3%), LLM-based chatbots (eg, ChatGPT, Bard, Gemini, YouChat, Copilot; 63/297, 21.2%), health applications (eg, diagnosis tool; 54/297, 18.2%), and conversational assistants (eg, Siri, Alexa, Google Home; 24/297, 24.7%). Additionally, 7 (2.4%) participants mentioned consulting other online sources such as academic journals and publications, government health websites, health charity websites, medical insurance carrier websites, MyChart (health records; Epic Systems Corporation), and podcasts. The mean number of source types (search engines, health-related websites, etc) per participant was 2.8 (SD 1.3). We did not find any significant differences based on the country of residence.

**Table 2:** Sources for online health information seeking (N=297).

Source	Values, n (%)
Search engines (eg, Google, Bing, and Yahoo)	291 (98)
Health-related websites (eg, WebMD, Mayo Clinic, and PubMed Central)	203 (68.4)
Social media (eg, Twitter, Facebook, Instagram, and Reddit)	119 (40.1)
Health community forums	84 (28.3)
LLM^a^-based chatbots (eg, ChatGPT, Bard, Gemini, YouChat, and Copilot; Microsoft Corp)	63 (21.2)
Health applications (eg, diagnosis tool)	54 (18.2)
Conversational assistants (eg, Siri, Alexa, and Google Home)	24 (24.7)
Government, charity, and insurance health websites	3 (1)
Academic journals and publications	2 (0.6)
Electronic health records (eg, MyChart)	1 (0.3)
Podcasts	1 (0.3)

^a^LLM: large language model.

Participants who consulted LLM-based chatbots for health information mentioned a wide range of chatbots they used in the prior year. The most popular LLM-based chatbot was ChatGPT (57/121, 47.1%), followed by Microsoft Copilot (19/121, 15.7%) and Gemini (18/121, 14.8%). Other chatbots used for online health information were Bard (9/121, 7.4%), YouChat (3/121, 2.5%), Claude (2/121, 1.7%), GPT-3 (2/121, 1.7%; OpenAI), Perplexity AI (2/121, 1.7%), Bing (1/121, 0.8%), Ginger (1/121, 0.8%), LaMDA (1/121, 0.8%; Google LLC), Leo (1/121, 0.8%; Leo AI, Inc), Mediktor (1/121, 0.8%), Orca (1/121, 0.8%; Microsoft Corp), Pi (1/121, 0.8%), Suki (1/121, 0.8%; Suki AI, Inc), and TechTarget (1/121, 0.8%).

### General ChatGPT Usage of Participants

To compare participants’ usage of LLM-based chatbots for health information searches and other uses, we investigated the familiarity and usage of ChatGPT, the most popular LLM-based chatbot for health information, in contexts outside of health information. The median value of participants’ familiarity with ChatGPT on a 5-point Likert scale (from “not familiar at all” to “extremely familiar”) was 3 (IQR 2). Most participants (230/297, 77.4%) reported that they used ChatGPT for various purposes in the past year. However, only 8.8% (26/230) had used the Pro (paid) version. A diverse range of languages was used with ChatGPT. The most popular natural language used by participants was English (215/230, 93.5%), followed by Spanish (17/230, 7.4%), German (11/230, 4.8%), Polish (11/230, 4.8%), French (6/230, 2.6%), and Portuguese (6/230, 2.6%).

Participants who have not used ChatGPT in the past year expressed several reasons for not using the technology in general. The most popular reason for not using ChatGPT was not finding the need to use it (34/74, 45.9%). Other reasons for nonuse include not trusting AI (11/74, 14.9%), not knowing what ChatGPT is or how to use it (4/74, 5.4%), ethical and privacy issues (4/74, 5.4%), negative prior experiences that did not provide satisfactory responses (3/74, 4.1%), and not wanting to make an account (2/74, 2.7%). Three participants mentioned that they preferred other ways of seeking information that were more familiar to them (3/74, 4.1%). Furthermore, 1 (1.4%) participant mentioned that they think it is a waste of resources, while another (1/74, 1.4%) participant mentioned preferring to use other AI chatbots instead of ChatGPT. Some participants even mentioned that they had not considered using ChatGPT at all (10/74, 13.5%).

### Topics of Health Information Sought Online

Participants sought several types of health information online (Table 3). Overall, 90.2% (268/297) of participants searched for information on health conditions or symptoms. The next popular topic was information on medications (179/297, 60.3%). Other topics included medical procedures or treatments (137/297, 46.1%), diet (132/297, 44.1%), fitness (119/297, 40.1%), and self-diagnosis (69/297, 23.2%). This breakdown did not differ significantly by participant country of residence.

**Table 3:** Topics of online health information sought by participant and source type. Multiple responses were allowed for each source.

Source type (number)	Health conditions or symptoms, n (%)	Medication, n (%)	Medical procedure or treatment, n (%)	Diet, n (%)	Fitness, n (%)	Self-diagnosis, n (%)
Overall, by participant^a^ (297)	268 (90.2)	179 (60.3)	137 (46.1)	132 (44.4)	119 (40.1)	69 (23.2)
Search engines (291)	248 (85.2)	148 (50.9)	102 (35.1)	105 (36.1)	91 (31.3)	58 (19.9)
Health-related websites (203)	164 (80.8)	83 (40.9)	57 (28.1)	27 (13.3)	19 (9.4)	18 (8.9)
Social media (119)	68 (57.1)	47 (39.5)	27 (22.7)	44 (37)	45 (37.8)	13 (10.9)
Health community forums (84)	63 (75)	29 (34.5)	27 (32.1)	18 (21.4)	16 (19)	11 (13.1)
LLM^b^-based chatbots (63)	34 (54)	17 (27)	18 (28.6)	21 (33.3)	21 (33.3)	13 (20.6)
Health applications (54)	26 (48.1)	14 (25.9)	5 (9.3)	10 (18.5)	20 (37)	6 (11.1)
Conversational assistants (24)	10 (41.7)	12 (50)	4 (16.7)	7 (29.2)	7 (29.2)	2 (8.3)

^a^Total number of unique topics searched online across all sources.

^b^LLM: large language model.

### Perceptions of Quality and Trust of Health Information From Online Sources

Across all sources, participants scored online health information as significantly more accurate, helpful, trustworthy, useful, and easy to understand than a neutral score of 3 on a 5-point scale from “strongly disagree” to “strongly agree” (Table 4). The median for all these characteristics was 4 (IQR 0-1). Participants were also satisfied with the online health information, as the median value was 4 (IQR 0-1). Considering how much the information reduced feelings of anxiety, there was more variability in the median values across the various sources of information. The median values for search engines and social media were the lowest with 3 (IQR 1) and 3.5 (IQR 1), respectively. For all other sources, the median values were slightly higher, being 4 (IQR 1).

**Table 4:** Perceptions and trust of health information from participants and their actions by source. Items on perceptions and trust of health information were on a 5-point Likert scale (from “strongly disagree” to “strongly agree”).

Source, name (number)	Accuracy, median (IQR)	Satisfaction, median (IQR)	Helpfulness, median (IQR)	Trustfulness, median (IQR)	Usefulness, median (IQR)	Easy to understand, median (IQR)	Reduced anxiety, median (IQR)	CCI^a^, n (%)	FIA^b^, n (%)
Search engines (291)	4 (0)	4 (0)	4 (0)	4 (1)	4 (0)	4 (0)	3 (1)	144 (49.5)	184 (63.2)
Health-related websites (203)	4 (1)	4 (1)	4 (0)	4 (1)	4 (1)	4 (0)	4 (1)	63 (31)	132 (65)
Social media (119)	4 (1)	4 (1)	4 (1)	4 (1)	4 (1)	4 (0)	3.5 (1)	43 (36.4)	59 (50)
Health community forums (84)	4 (0)	4 (0)	4 (1)	4 (0)	4 (0)	4 (1)	4 (1)	19 (22.6)	49 (58.3)
LLM^c^-based chatbots (63)	4 (0)	4 (1)	4 (0)	4 (1)	4 (0)	4 (1)	4 (1)	12 (19.4)	30 (48.4)
Health applications (54)	4 (0)	4 (1)	4 (0)	4 (0.25)	4 (0)	4 (1)	4 (1)	6 (11.3)	31 (58.5)
Conversational assistants (24)	4 (1)	4 (1)	4 (0)	4 (1)	4 (0)	4 (0.25)	4 (1)	3 (12.5)	10 (41.7)

^a^CCI: cross-checked information.

^b^FIA: followed information or advice.

^c^LLM: large language model.

We also investigated how often participants reported cross-checking information obtained from different types of online sources. Participants most frequently cross-checked information obtained from search engines, with 49.5% (144/291) verifying it against other sources. In comparison, 36.4% (43/119) cross-checked information from social media, 31% (63/203) from health-related websites, 22.6% (19/84) from health community forums, 19.4% (12/63) from LLM-based chatbots, 12.5% (3/24) from conversational assistants, and 11.3% (6/54) from health applications.

We also investigated how often participants reported following the health care advice they obtained online by source type. Health-related websites had the highest rate of reported adherence, with 65% (132/203) of participants following the advice obtained, followed by search engines at 63.2% (184/291), health applications at 58.5% (31/54), health-community forums at 58.3% (49/84), and social media at 50% (59/119). In contrast, the lowest adherence rates were found for LLM-based chatbots and conversational assistants, with 48.4% (30/63) and 41.7% (10/24) of participants following the advice, respectively.

Table 5 outlines the common methods and sources participants used to cross-check information found online. For information obtained via search engines, the most frequent cross-checking method was consulting health care professionals (40/132, 30.3%). Other widely used methods across various sources included referencing reputable medical and health websites (eg, WebMD, Mayo Clinic, National Institutes of Health, NHS), reviewing multiple sources from search engine results, and consulting research articles on platforms such as PubMed or Google Scholar. Although less common, a small percentage of participants (5/214, 2.3%) reported using AI tools to verify information from search engines and health-related websites.

**Table 5:** Methods used by participants to cross-check health information found online from various sources.

Source type	Number	Methods or sources for cross-checking (n)
Search engines	132	Health care professionals (40), reliable sources such as official health websites and government sites (28), use another search engine for results (18), research papers found in PubMed or Google Scholar (13), social media—Twitter, TikTok, and Reddit (12), health forums and experiences of others (8), AI^a^ tools (4), online health apps (2), Wikipedia (2), media such as YouTube or podcasts (3), health charity website (1), and paper that came with medication (1)
Health-related websites	82	Reputable medical and health websites such as WebMD, Mayo Clinic, NHS, patient resource site, government health websites (20), health care professionals (19), multiple sources and websites (19), medical or scientific studies from PubMed, Nature, Lancet, or Google Scholar (11), use another search engine for results (4), check for credentials of sources (3), social media—Reddit (2), AI tool (1), ask family member (1), medical lectures (1), and Wikipedia (1)
Social media	24	Consult health care professionals (8), various sources from Google search (6), reputable sources such as PubMed and NIH^b^ (4), nonspecific other websites (3), audiobooks (1), books (1), and social media doctors (1)
Health community forums	26	Various websites from Google search (8), reputable medical and health websites (7), health care professionals (4), academic studies from PubMed (2), other reputable health forums (1), product reviews (1), reference book (1), social media (1), and using TrustPilot to check the reliability of site and information (1)
LLM^c^-based chatbots	16	Various sources from Google search (6), reputable medical and health websites such as NIH (6), professionally written publications such as papers and books (3), and podcasts (1)
Health applications	4	Reputable and trusted websites such as NIH (2) and personal doctor (2)
Conversational assistants	3	Personal doctor (2) and health forums (1)

^a^AI: artificial intelligence.

^b^NIH: National Institutes of Health.

^c^LLM: large language model.

### Bivariate Analysis Results

Table 6 presents correlations between participants’ sociodemographic characteristics and their online health information–seeking behaviors. Age showed a negative correlation with the use of LLM-based chatbots for health information (ρ=–0.16, *P*=.006), while having a chronic health condition was positively correlated with the number of source types consulted (ρ=0.23, *P*<.001). eHEALS scores correlated positively with the number of source types used (ρ=0.23, *P*<.001), following the information (ρ=0.23, *P*=.02), and cross-checking information (ρ=0.12, *P*=.04). Additionally, AIAS-4 scores had significant positive correlations with all online health information–seeking behavior variables except cross-checking. Notably, participants’ positive perceptions of AI’s benefits for medicine were positively correlated with the number of source types used (ρ=0.14, *P*=.01), use of LLM-based chatbots (ρ=0.31, *P*<.001), and the number of health topics searched for (ρ=0.19, *P*<.001). Familiarity with ChatGPT was also significantly correlated with the number of source types consulted (ρ=0.16, *P*=.01), LLM-based chatbot use (ρ=0.30, *P*<.001), and the number of health topics searched for (ρ=0.24, *P*<.001). No significant relationships were found between online health information–seeking behaviors and years of education or T-HCT scores.

**Table 6:** Spearman correlations between sociodemographic characteristics of participants and their online health information–seeking behaviors.

Characteristics		Number of source types^a^			Usage of LLM^b^-based chatbot for health informations^c^					Number of topics searched^a^					Cross-checking information^d^					Following information and advice^d^		
		ρ		*P* value	ρ		*P* value		ρ			*P* value		ρ			*P* value		ρ			*P* value
Age (years)		–0.1		.1	–0.16		.006		–0.11			.06		0.11			.07		0.12			.05
Education (years)		–0.02		.67	–0.06		.33		0.02			.68		–0.01			.82		–0.01			.86
Chronic health condition status		0.23		<.001	–0.01		.81		0.1			.09		0.02			.79		–0.05			.37
T-HCT^e^		–0.07		.23	–0.06		.32		–0.09			.13		0.02			.76		0.08			.21
eHEALS^f^		0.23		<.001	0.05		.38		0.11			.07		0.12			.04		0.14			.02
**Artificial Intelligence Attitude Scale (AIAS)**																						
	AIAS-4^g^	0.15	.01		0.36	<.001		0.18			.002		–0.13			.03		0.13			.04	
	Medicine	0.14	.01		0.31	<.001		0.19			<.001		–0.06			.31		0.08			.16	
Familiarity with ChatGPT		0.16		.01	0.3		<.001		0.24			<.001		0.01			.83		0.04			.55

^a^Sum of all reported by the participant.

^b^LLM: large language model.

^c^Coded as 0 if the participant reported not using large language model–based chatbots for health information and 1 if they have used them.

^d^Average across all sources reported by the participant. Values range from 0 to 1.

^e^T-HCT: Trust in the Health Care Team.

^f^eHEALS: eHealth Literacy Scale.

^g^AIAS-4: 4-item Artificial Intelligence Attitude Scale.

## Discussion

### Principal Findings

This study provides insights into the evolving landscape of online health information–seeking behaviors by international online crowd workers, with a particular focus on the growing role of LLM-based chatbots in comparison to traditional online sources. The results highlight that while nearly all participants relied on search engines and health-related websites (291/297, 98% and 203/297, 68.4%, respectively) for health information, a notable proportion (63/297, 21.2%) also used LLM-based chatbots, such as ChatGPT, within the prior year. These results align with prior research on how patients and consumers search for health information online [6,7] while also offering more granular insights into chatbot usage.

As expected, ChatGPT was the most frequently consulted chatbot, though some participants also reported using other LLM-based tools, including Microsoft Copilot, Gemini, Bard, and YouChat. However, access to these technologies may not be uniform. Subscription-based models can limit availability to advanced chatbot versions, potentially exacerbating disparities in health information access. Those who could benefit most from these tools may face financial or technological barriers, restricting engagement and reducing the potential impact of LLM-based chatbots for underserved populations. Our findings showed that a very small number of participants used paid AI chatbots such as Ginger and Mediktor.

Considering the health topics that were searched for online, most participants sought information on health conditions or symptoms, followed by medications, medical procedures or treatments, diet, fitness, and self-diagnosis. However, when we look at the breakdown across sources, we see different patterns. LLM-based chatbots were consulted for self-diagnosis more frequently than any of the other sources of online health information. Additionally, medication queries were low with LLM-based chatbots compared to other popular sources, while diet and fitness were higher.

Our results did not show significant differences in participants’ perceptions of the quality and trust of health information across sources. Information from LLM-based chatbots was not rated significantly lower or higher in quality than other sources. However, participants were more cautious in their use of LLM-based chatbots for health information compared to other sources. Although 230/297 (77.4%) of participants used LLM-based chatbots for general purposes, only a fraction of them consulted them for health or medical information in the past year. Furthermore, only 19.4% (12/63) of participants cross-checked the information, and 48.4% (30/63) of participants reported following the advice obtained from these tools. Both rates were lower than search engines, health-related websites, forums, or social media.

Our qualitative analysis revealed that respondents used similar methods to cross-check online health information, regardless of source. Popular methods for verification included consulting health care professionals, cross-referencing with reputable health websites, or referencing research papers from academic sources. Although fewer unique methods of cross-checking were reported for verifying information from LLM-based chatbots, they followed similar patterns to those of more traditional sources, such as search engines and health-related websites. These cross-checking practices highlight a general awareness among participants of the need for accuracy in health-related decisions, especially when interacting with online sources that may suggest misinformation or inaccurate advice.

Finally, this study identified key correlations between sociodemographic factors and information-seeking behavior variables. Age was negatively correlated with LLM-based chatbot use, suggesting that younger participants were more comfortable exploring novel, AI-driven platforms for health information. Having a chronic health condition was positively correlated with the number of information source types consulted, indicating that individuals managing ongoing health issues may seek out diverse viewpoints and platforms to support their health decisions. Higher eHealth literacy was also associated with more active engagement, such as cross-checking and adhering to health advice, reflecting the importance of digital literacy in navigating a variety of online resources effectively. Additionally, a higher positive attitude toward AI was correlated with LLM-based chatbot use, cross-checking, and following the information. This result highlights the importance of attitudes toward AI determining the adoption of AI-based tools even in high-risk contexts such as health information searching.

While LLM-based chatbots are increasingly being used for health information, participants’ low rate of both cross-checking and adherence to the information may hint at their utility, especially when compared to more established online health sources. At the same time, LLM-based chatbots offer notable advantages, such as generating human-like conversations with remarkable fluency and coherence, making interactions more engaging and user-friendly [35]. Unlike traditional search engines that return a list of relevant web pages, these chatbots can provide direct answers to users’ queries, creating a more personalized and user-engaging search experience [36]. Prior research suggests that LLM-based chatbots can also reduce users’ cognitive load, further improving their overall experience and satisfaction [37]. However, this study highlights a need for improvements in LLM-based chatbot accuracy and transparency, as well as the importance of digital literacy in supporting responsible health information–seeking behaviors. As LLM-based technologies continue to evolve, these results provide important insights for motivating the need for designing AI tools that are more accurate, transparent, and useful to support users in safely navigating health information online.

### Limitations

Several limitations may impact the generalizability of the findings reported in this paper. Most importantly, some of our findings may be biased due to self-selection by respondents and a small sample. Our participants were recruited from a web-based crowdwork platform and, therefore, are usually more comfortable and willing to use technology than other samples of the population. Additionally, our study lacks regional language factors of using LLM-based chatbots. We only included participants who use English as a primary language despite recruiting participants internationally. Further, we did not collect data on language preferences for using chatbots such as ChatGPT. Furthermore, the survey is cross-sectional and may not reflect changes that accompany releases of new and better LLMs available to the public, as technology in this space evolves rapidly.

### Conclusions

In this study, we investigated the prevalence of LLM-based chatbot use for health information in comparison to other internet-based sources. Additionally, we investigated the trust respondents place in this information and their subsequent actions to cross-check and follow the information received. Our results highlight the growing presence of LLM-based chatbots in online health information search behaviors, revealing that while traditional sources such as search engines and health websites remain dominant, chatbots are emerging as a noteworthy resource for some users, specifically those who are younger and find AI to be more trustworthy. We did not find any significant differences in online health information–seeking behaviors based on the countries of residence of the participants. Additionally, there were no significant quantitative differences in perceived trust and quality of the health information or the methods of cross-checking across sources. However, the use of LLM-based chatbots for health information and the rate of cross-checking or following the advice from these tools seemed more cautious. As LLMs continue to evolve, enhancing their accuracy and transparency will be essential in mitigating any potential risks by supporting responsible information-seeking and maximizing the potential of AI in health contexts.

## Appendix

Multimedia Appendix 1 Survey instrument.

## Abbreviations

AI: artificial intelligence
AIAS-4: 4-item Artificial Intelligence Attitude Scale
eHEALS: eHealth Literacy Scale
LLM: large language model
OECD: Organisation for Economic Co-Operation and Development
T-HCT: Trust in the Health Care Team

## Data Availability

The anonymized data analyzed in this study can be obtained from the corresponding author upon reasonable request.
