# Supplementary material for: Online Health Information–Seeking in the Era of Large Language Models: Cross-Sectional Web-Based Survey Study
Source: J Med Internet Res. 2025 Mar 31;27:e68560. doi: 10.2196/68560 (PMC11997521; doi:10.2196/68560)
Supplement: Multimedia Appendix 1 [file jmir_v27i1e68560_app1.doc]

**This is a Multimedia Appendix to a full manuscript published in the J Med Internet Res. For full copyright and citation information see http://dx.doi.org/10.2196/jmir.68560**

Start of Block: Sociodemographics

Do you speak English as a native/first language?

- Yes
- No (enter your native language below) __________________________________________________

How many years of education have you completed? 

Please include the total number of years you have completed primary/elementary, secondary, and/or post-secondary education.

________________________________________________________________

What is your occupation? If you are retired or otherwise not working, please let us know.

________________________________________________________________

Based on your country of residence's average household net adjusted disposable income, what best describes your total yearly net household income?

 Use the following link to find the average for your country of residence: https://www.oecdbetterlifeindex.org/topics/income/

- Extremely below the median
- Somewhat below the median
- Around the median
- Somewhat above the median
- Extremely above the median

Are you diagnosed with at least one chronic health disease or condition?

- Yes
- No
- Prefer not to answer

End of Block: Sociodemographics

Start of Block: T-HCT

The following questions ask for your **opinions and beliefs about people who work in health care**, such as doctors, nurses, pharmacists, physician assistants, and administrative staff.

These individuals work in the health care system. The health care system includes places like medical clinics, hospitals, and health insurance companies.

Please think about your general impressions of the health care system and people who work in health care. These impressions might be based on your experiences or things you have heard from family, friends, or the media. Please indicate **how much you disagree or agree with each of the statements**.

|  | Strongly Disagree | Disagree | Neither Agree nor Disagree | Agree | Strongly Agree |
| --- | --- | --- | --- | --- | --- |
| People who work in health care have good judgment. |  |  |  |  |  |
| People who work in health care explain the benefits and risks of treatments to patients. |  |  |  |  |  |
| People who work in health care listen to patients. |  |  |  |  |  |
| People who work in health care believe patients when they say something is wrong. |  |  |  |  |  |
| People who work in health care follow up with patients when needed. |  |  |  |  |  |
| People who work in health care put making money above patient needs. |  |  |  |  |  |
| People who work in health care recommend expensive treatments to make money. |  |  |  |  |  |
| People who work in health care hide mistakes. |  |  |  |  |  |
| People who work in health care might experiment on patients without their knowledge. |  |  |  |  |  |
| People who work in health care rush through appointments. |  |  |  |  |  |
| People who work in health care are held accountable if they make a mistake. |  |  |  |  |  |
| People who work in health care are held accountable if they treat patients unfairly. |  |  |  |  |  |
| People who work in health care are held accountable if they discriminate against patients. |  |  |  |  |  |
| People who work in health care keep medical records private. |  |  |  |  |  |
| People who work in health care use secure systems to store medical records. |  |  |  |  |  |
| People who work in health care respect patient privacy. |  |  |  |  |  |
| People who work in health care treat patients fairly, regardless of their ability to pay. |  |  |  |  |  |
| People who work in health care treat patients of all races and ethnicities fairly. |  |  |  |  |  |
| People who work in health care treat patients fairly, regardless of their gender (e.g., male, female, or nonbinary). |  |  |  |  |  |
| People who work in health care treat patients fairly, regardless of their sexual orientation (e.g., straight, gay, lesbian, or bisexual). |  |  |  |  |  |
| People who work in health care treat patients fairly, regardless of their weight. |  |  |  |  |  |
| People who work in health care treat patients fairly, regardless of their religion. |  |  |  |  |  |
| People who work in health care treat patients fairly, regardless of their education level. |  |  |  |  |  |
| People who work in health care treat patients with a history of mental illness unfairly. |  |  |  |  |  |
| People who work in health care treat patients diagnosed with HIV unfairly. |  |  |  |  |  |
| People who work in health care treat patients who abuse drugs unfairly. |  |  |  |  |  |
| All things considered, I trust people who work in health care. |  |  |  |  |  |
| I put my trust in people who work in health care. |  |  |  |  |  |
| People who work in health care are trustworthy. |  |  |  |  |  |

End of Block: T-HCT

Start of Block: eHealth Literacy

I would like to ask you for your opinion and about your experience using the Internet for health information. For each statement, tell me which response best reflects your opinion and experience *right now*.

How **useful** do you feel the Internet is in helping you in making decisions about your health?

- Not useful at all
- Not useful
- Unsure
- Useful
- Very Useful

How **important** is it for you to be able to access health resources on the Internet?

- Not important at all
- Not important
- Unsure
- Important
- Very important

I know **what** health resources are available on the Internet

- Strongly Disagree
- Disagree
- Undecided
- Agree
- Strongly Agree

I know **where** to find helpful health resources on the Internet

- Strongly Disagree
- Disagree
- Undecided
- Agree
- Strongly Agree

I know **how** to find helpful health resources on the Internet

- Strongly Disagree
- Disagree
- Undecided
- Agree
- Strongly Agree

I know **how to use** the Internet to answer my questions about health

- Strongly Disagree
- Disagree
- Undecided
- Agree
- Strongly Agree

I know how to use **the health information** I find on the Internet to help me

- Strongly Disagree
- Disagree
- Undecided
- Agree
- Strongly Agree

I have the skills I need to **evaluate** the health resources I find on the Internet

- Strongly Disagree
- Disagree
- Undecided
- Agree
- Strongly Agree

I can tell **high quality** health resources from **low quality** health resources on the Internet

- Strongly Disagree
- Disagree
- Undecided
- Agree
- Strongly Agree

I feel **confident** in using information from the Internet to make health decisions

- Strongly Disagree
- Disagree
- Undecided
- Agree
- Strongly Agree

End of Block: eHealth Literacy

Start of Block: AIAS-4

Below you will find sentences about the attitude toward Artificial Intelligence (AI).

1 = Not at all
10 = Completely agree

|  | 1 | 2 | 3 | 4 | 5 | 6 | 7 | 8 | 9 | 10 |
| --- | --- | --- | --- | --- | --- | --- | --- | --- | --- | --- |
| I believe that AI will improve my life |  |  |  |  |  |  |  |  |  |  |
| I believe that AI will improve my work |  |  |  |  |  |  |  |  |  |  |
| I think I will use AI technology in the future |  |  |  |  |  |  |  |  |  |  |
| I think AI technology is positive for humanity |  |  |  |  |  |  |  |  |  |  |
| I think AI can have a beneficial impact in medicine |  |  |  |  |  |  |  |  |  |  |

End of Block: AIAS-4

Start of Block: Health Information Search

Over the last year, what tools have you used to search online for health-related information?

- search engine (Google, Bing, Yahoo, etc.)
- social media (Twitter, Facebook, Instagram, Reddit, etc)
- health community forum
- health-related website (e.g., WebMD, Mayo Clinic, PubMed Central, etc)
- large language model-based chatbot (e.g., ChatGPT, Bard, Gemini, Microsoft Copilot, YouChat, Perplexity AI, ErnieBot, etc.)
- conversational assistant (e.g., Siri, Alexa, Google Home)
- health app (e.g., diagnosis tool)
- other __________________________________________________

Display This Question:

If Over the last year, what tools have you used to search online for health-related information? = large language model-based chatbot (e.g., ChatGPT, Bard, Gemini, Microsoft Copilot, YouChat, Perplexity AI, ErnieBot, etc.)

Which large language model-based chatbot(s) (e.g., ChatGPT, Bard, Gemini, Microsoft Copilot, YouChat, Perplexity AI, ErnieBot, etc.) have you used? List all that applies with a comma in between each chatbot.

________________________________________________________________

End of Block: Health Information Search

Repeat this block of questions for each response about the sources above

Start of Block: Trust and Perceptions

Think about the most recent time you can recall using a ${source name} to search for health information online.

What were you searching for?

- Information about a health condition/symptoms
- Information about a medication
- Information about a medical procedure or treatment
- Information about fitness
- Information about diet
- Self-diagnosis
- Other __________________________________________________

For each statement, which response best reflects your opinion and experience using a ${source name} to search health information.

|  | Strongly disagree | Disagree | Undecided | Agree | Strongly agree |
| --- | --- | --- | --- | --- | --- |
| I found the information accurate. |  |  |  |  |  |
| I was satisfied with the information I received. |  |  |  |  |  |
| I found the information I received helpful for my context. |  |  |  |  |  |
| I trusted the information and results I received. |  |  |  |  |  |
| I found the information I received useful for my context. |  |  |  |  |  |
| I found the information easy to understand. |  |  |  |  |  |
| The information I received made me feel less anxious. |  |  |  |  |  |

Did you act on or actually follow the information you received from a ${source name}?

- No
- Yes
- Unsure

Did you cross check the information you received from a ${lm://Field/1}?

- No
- Yes
- Unsure

Display This Question:

If Did you cross check the information you received from a ${lm://Field/1}? = Yes

How did you cross check the information? Where did you go to cross check?

________________________________________________________________

End of Block: Trust and Perceptions

Start of Block: ChatGPT

How familiar are you with ChatGPT?

- Not familiar at all
- Slightly familiar
- Moderately familiar
- Very familiar
- Extremely familiar

In the past year, have you used ChatGPT for anything?

- Yes
- No

Display This Question:

If In the past year, have you used ChatGPT for anything? = Yes

In the past year, have you used the paid Pro version?

- Yes
- No
- I don't know what the paid Pro version is

Display This Question:

If In the past year, have you used ChatGPT for anything? = Yes

In the past year, what language(s) did you use when you used ChatGPT? List all that applies with a comma in between each language.

________________________________________________________________

Display This Question:

If In the past year, have you used ChatGPT for anything? = No

What are your reasons for not using ChatGPT?

________________________________________________________________

End of Block: ChatGPT
